# Development of Electrospun Chitosan-Polyethylene Oxide/Fibrinogen Biocomposite for Potential Wound Healing Applications

**DOI:** 10.1186/s11671-018-2491-8

**Published:** 2018-04-02

**Authors:** Tony T. Yuan, Ann Marie DiGeorge Foushee, Monica C. Johnson, Angela R. Jockheck-Clark, Jonathan M. Stahl

**Affiliations:** Naval Medical Research Unit San Antonio, 3650 Chambers Pass, Bldg 3610 BHT-2, JBSA Fort Sam Houston, TX 78234-6315 USA

**Keywords:** Electrospinning, Biomedical applications, Chitosan, Wound dressing, Fibrinogen, Nanofibers, PDGF, Wound healing

## Abstract

Normal wound healing is a highly complex process that requires the interplay of various growth factors and cell types. Despite advancements in biomaterials, only a few bioactive wound dressings reach the clinical setting. The purpose of this research was to explore the feasibility of electrospinning a novel nanofibrous chitosan (CS)-fibrinogen (Fb) scaffold capable of sustained release of platelet-derived growth factor (PDGF) for the promotion of fibroblast migration and wound healing. CS-Fb scaffolds were successfully electrospun using a dual-spinneret electrospinner and directly evaluated for their physical, chemical, and biological characteristics. CS-polyethylene/Fb scaffolds exhibited thinner fiber diameters than nanofibers electrospun from the individual components while demonstrating adequate mechanical properties and homogeneous polymer distribution. In addition, the scaffold demonstrated acceptable water transfer rates for wound healing applications. PDGF was successfully incorporated in the scaffold and maintained functional activity throughout the electrospinning process. Furthermore, released PDGF was effective at promoting fibroblast migration equivalent to a single 50 ng/mL dose of PDGF. The current study demonstrates that PDGF-loaded CS-Fb nanofibrous scaffolds possess characteristics that would be highly beneficial as novel bioactive dressings for enhancement of wound healing.

## Background

Despite numerous advances made in the field of wound management, few bioactive dressings have been commercialized with the capacity to enhance the healing process. This lack of successful product can be attributed to the complexity of the wound healing process, which involves numerous soluble mediators, blood cells, parenchymal cells, and extracellular matrix (ECM) components. Growth factors play a central role in the wound healing process by promoting cell proliferation, migration, and differentiation. However, rapid elimination and short half-lives in the body [1–3] have limited the clinical adoption of growth factor therapy for promotion of healing and tissue regeneration [4, 5]. Currently, repetitive in vivo administration of growth factors is required to achieve therapeutic effects (e.g., cellular recruitment and differentiation). The development of a wound dressing that is capable of sustained, localized growth factor delivery would significantly improve treatment outcomes and accelerate clinical adoption.

Platelet-derived growth factor (PDGF) plays a critical role as an initiator and mediator of wound healing; it acts as a chemotactic agent for neutrophils, monocytes, and fibroblasts [6] and helps regulate matrix deposition. In addition, PDGF can inhibit differentiation of fibroblasts into myofibroblasts, which can impact wound contractions and decrease scar formation [6]. PDGF, sold under the brand name Regranex®, is the only growth factor currently approved by the FDA and is intended for the treatment of lower extremity diabetic ulcers. Once daily topical application of Regranex® gel (2.2 μg/cm^2^ of ulcer) has been shown to result in a 30% faster healing time with minimal adverse effects. However, daily topical application and removal of Regranex® is inconvenient and can adversely impact patient quality of life.

Electrospinning has been established as a notable technique in the fabrication of biomimetic nanofibrous scaffolds using biologically significant polymers. Numerous biodegradable synthetic (e.g., polycaprolactone) and natural polymers (e.g., collagen) have been electrospun into non-woven nanofibrous scaffolds that demonstrate high surface area-to-volume ratios and porosity characteristics, which are important for drug delivery and wound dressing applications. Naturally occurring polymers (e.g., collagen, elastin, and fibrinogen) are particularly attractive wound dressing materials due to their bioactivity, biocompatibility, and unique ability to bind specific growth factors, such as PDGF. Fibrinogen (Fb), a 340-kDa globular plasma protein, plays a major role in clot formation through its activated form, fibrin, which functions as a temporary matrix for tissue repair and regeneration. The absence of Fb and fibrin has been correlated with wound healing defects [7]. Fb-based products demonstrate biodegradability, non-immunogenicity, and cellular migration promotion and have been previously developed as hydrogels [8–10] and wet extrusion cables [11, 12]; however, the physical and structural properties of these materials have limited their clinical adoption. Hydrogels lack structural and mechanical integrity for long-term use, and wet extrusion fibers exhibit exponentially larger diameter sizes (200–250 μm) than the native ECM (200–500 nm). Wnek et al. demonstrated that electrospinning, however, offers the capability to fabricate Fb scaffolds that closely resemble natural ECM structures [13]. Although the resulting nanofibers exhibited improved mechanical properties over hydrogels, the structure still lacked optimum mechanical properties for wound dressing applications [14].

To improve the mechanical properties of electrospun fibrinogen scaffold, additional polymer could be introduced to strengthen the nanofibers [15]. Specifically, chitosan (CS), an *N*-deacetylated derivative of chitin, has been shown to produce desirable antimicrobial properties in wound dressing applications [16, 17]. In addition to being a potent antimicrobial, CS has been electrospun for various biomedical applications including guided bone regeneration [18, 19], transdermal drug delivery [20], directed stem cell differentiation [21], and hemostat [22]. Due to the polycationic nature of chitosan, there are significant difficulties in electrospinning using common solvents. To overcome these difficulties and improve electrospinninability, CS has been mixed with various other polymers such as poly(vinyl pyrrolidone) [23], poly(ethylene oxide) (PEO) [16], and poly(vinyl alcohol) [24]. Similarly, the polycationic nature of CS has limited successful combination with Fb into a single scaffold due to strong electrostatic interactions. In this work, this limitation was overcome by utilizing a versatile dual-nozzle electrospinning system. The resultant nanofibrous scaffolds were analyzed using various physiochemical characterizations. We hypothesized that the novel combinatory CS-Fb nanofibrous scaffold would present a viable candidate for a bioactive and biomimetic wound dressing that could deliver PDGF and could be effective in influencing fibroblast migration required for wound healing.

## Methods

### Materials

Acetic acid (glacial, ≥ 99.85%), CS (low molecular weight, 75–85% deacetylation), PEO (MW 300,000 g/mol), Fb (type I-S, 65–85% protein), 1,1,1,3,3,3-hexafluoroisopropanol (HFIP), and bovine serum albumin (BSA, ≥ 96%) were purchased from Sigma-Aldrich (St. Louis, MO, USA). Dimethyl sulfoxide (DMSO) was purchased from EMD Millipore (Billerica, MA, USA). Human dermal fibroblasts (PCS-201-012) and cell culture reagents were purchased from American Type Culture Collection (Manassas, VA, USA). Carrier-free recombinant human platelet-derived growth factor-BB (rhPDGF-BB) was purchased from PeproTech (Rocky Hill, NJ, USA). All reagents were of reagent grade and used without further purification or modification.

### Preparation of Electrospun Chitosan-Polyethylene Oxide/Fibrinogen Composite Scaffolds

**Fig. 1:**
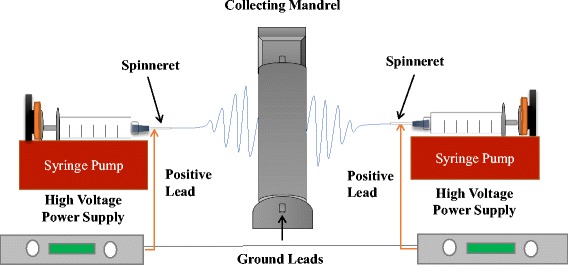
Schematic of dual-spinneret electrospinner

Chitosan-PEO stock solutions were made by dissolving CS and PEO in 1% acetic acid with BSA (0.5% *v*/*v*) and DMSO (10% *v*/*v*) to obtain a total polymer content of 5.5 wt.% with a mass ratio of 2:1 CS to PEO. Polyethylene oxide was added to improve the ability to electrospin CS as described previously [16]. Fibrinogen stock solutions (110 mg/mL) were made by dissolving Fb in one-part 10× Eagle’s minimum essential media (EMEM) and nine parts HFIP. CS-PEO and Fb stock solutions were stirred at room temperature until completely dissolved. For loaded scaffolds, PDGF was mixed into each polymer solution immediately before electrospinning. Electrospun CS-PEO/Fb composite nanofibrous scaffolds were fabricated using a multi-nozzle electrospinning system (Fig. 1). Solutions were individually loaded and mechanically pumped into the system using a disposable syringe, connected to an 18-gauge spinneret and NE-1000 syringe pump (New Era Pump Systems, Farmingdale, NY), at a flow rate of 0.7 and 1.0 mL h^−1^ for CS-PEO and Fb, respectively. The mandrel collector, rotating at 25 RPM, was located between the spinneret tips at a distance of 22 cm for CS-PEO and 12.5 cm for Fb. During the fabrication process, DC voltages of 28 and 22 kV were applied to the spinneret tips for CS-PEO and Fb polymer solutions, respectively. Fabricated nanofiber scaffolds were either collected on 18-cm glass coverslips for biological assays or directly cut from the mandrel for permeability and tensile strength analysis. All electrospinning experiments were performed at room temperature and a relative humidity of 35–45% for 3 h. Samples containing PDGF were stored at − 20 °C in a desiccator, while unloaded scaffolds were stored in a desiccator at room temperature after fabrication.

### Nanofiber Morphology and Diameter

Electrospun nanofibrous scaffolds were sputter-coated with gold and observed using field emission scanning electron microscopy (FESEM) (Zeiss Sigma VP-40, Germany). Twenty micrographs were taken per scaffold, and three individually spun scaffolds were analyzed per formulation. Mean nanofiber diameters were calculated by measuring five random points per micrograph using ImageJ (NIH, Bethesda, MD). A total number of 100 measurements were made per scaffold formulation.

### Surface Chemistry Characterization

Time-of-flight ion mass spectrometry (ToF-SIMS) was used to analyze the surface chemistry and the polymer component contributions in the scaffold. ToF-SIMS is a sensitive and in-depth surface analytical methodology which assesses the chemical composition of the top nanometer layer of a material by bombardment with high-energy Bi_3_^2+^ clusters under ultra-high vacuum [25, 26]. Secondary ion fragments ejected from the material surface are then analyzed based on their atomic mass to charge (m/z) ratios and used to infer the overall surface chemical composition.

ToF-SIMS analysis was performed by Evans Analytics Group (EAG, Sunnyvale, CA) using a ToF-SIMS IV (Ion-ToF GmBH, Germany) with a bismuth liquid metal ion gun (30 kV). The instrument was operated in an ion microprobe mode where three positive and negative ion spectra were acquired from each sample. Selected positive and negative ions were mapped over a 50 × 50-μm raster area with a 2048 × 2048-pixel image resolution to provide polymer component mapping of the scaffolds. A normalized intensity, defined as the count of each secondary ion divided by the total count of ions recorded multiplied by 10,000, was reported for each fragment. The mean of three different locations on the surface of six independently fabricated samples was analyzed (*n* = 6). CS-PEO and Fb polymer distribution analyzed by ToF-SIMs was representative of the whole scaffold.

Water contact angles of CS-PEO, CS-PEO/Fb, and Fb scaffolds were determined in accordance with the American Standards for Testing Methods (ASTM) D7334-08. Nanofibrous scaffolds were electrospun onto glass coverslips. A single droplet of distilled water (2 μL) was applied to the surface of the scaffold and the contact angle measured within 30 s using Krüss DSA10 MK2 Drop Shape Analyzer (Krüss, Hamburg, Germany). The measurements were repeated three times at different locations for each sample, and the mean contact angle was calculated. Six independently electrospun scaffolds were analyzed (*n* = 6).

### Mechanical Uniaxial Tensile Strength

The mechanical properties of electrospun scaffolds were measured at room temperature using an Instron® E3000 ElectroPuls (Instron, Canton, MA) equipped with a 250-N load cell at a strain rate of 1 mm min^−1^. The thickness of the samples was measured at six random positions by Keyence 3D Laser Scanning Microscope VK-200 (Keyence, Itasca, IL). The thickness of the scaffolds used for tensile testing was 93.8 ± 7.4 μm. Before the test, the scaffolds were cut into 40 × 25-mm rectangular specimens. Data obtained were reported and plotted as stress-strain curves, where tensile stress was defined as the ratio of force to the cross-sectional area of the specimen and where the strain was defined as the change in length over the original length. Young’s moduli, tensile stress, and strain-at-break calculations were obtained from stress-strain curves from an average of ten samples per formulation.

### Water Vapor Transfer Rate

Water vapor transfer rate of CS-PEO/Fb scaffolds was measured as per the ASTM E96/E96M desiccant method. A total of six independently electrospun specimens were prepared per formulation and placed on the opening of test vessels containing reusable silica gel desiccant at room temperature and a relative mean humidity of 58.2 ± 5.2%. Water vapor transmission rate (WVTR) was calculated from the weight of the test containers measured at different time points over 48 h:

1 $$ \mathrm{WVTR}=\frac{G}{t\times A} $$

where *G* represents the change in weight of the test container, *t* is elapsed time, and *A* is the cross-sectional area of the scaffold.

### In Vitro Cell Viability

Indirect cytotoxicity of the scaffolds was evaluated based on an approach adapted from the ISO10993-5 standard test method [27, 28]. Human dermal fibroblasts were cultured at 37 °C and 5% CO_2_ in serum-free fibroblast media and refreshed every 3 days. Once the cells reached confluence, they were trypsinized and seeded into 12-well plates (10,000 cells/mL). The following day, media were replenished and nanofibrous scaffolds were introduced. Cell proliferation was monitored over 120 h using a 2-(4-iodophenyl)-3-(4-nitrophenyl)-5-(2,4-disulfophenyl)-2H-tetrazolium sodium (WST-1) cell proliferation assay.

### Visualization of Fibroblasts

Human dermal fibroblasts were trypsinized and seeded onto CS-PEO, Fb, and CS-PEO/Fb scaffolds. After 24 h of incubation at 37 °C and 5% CO_2_, the cells were washed and stained with LIVE/DEAD™ cell viability kit (ThermoFisher Scientific, Waltham, MA, USA) according to manufacturer’s specifications. Additionally, adhesion and attachment of human fibroblasts to the scaffold were evaluated by staining with Phalloidin-Atto 565 (Sigma Aldrich and 4′,6-diamidino-2-phenylindole dihydrochloride (DAPI; ThermoFisher Scientific) according to manufacturer’s specifications. Images were observed and taken using an inverted confocal microscope (Nikon C1, C1EZ, Melville, NY, USA).

### In Vitro Degradation

The degradation of CS-PEO/Fb nanofibrous scaffolds was performed in fibroblast basal medium (FBM, ATCC) at 37 °C and 5% CO_2_. Scaffolds were immersed in FBM and incubated for 1, 6, 24, or 48 h. The initial dry weight of each scaffold was noted; at each time point, the scaffolds were washed, freeze-dried, and weighed again. The degradation of the scaffold was calculated from the following formula:

2 $$ \mathrm{Degradation}\%=\left({W}_0-{W}_{\mathrm{t}}\right)/{W}_0\times 100 $$

where *W*_0_ is the initial weight of the scaffold, and *W*_t_ is the weight of the scaffold at respective time point.

### PDGF Release and Detection

Eluates, collected from specific time points during in vitro degradation experiment, were assayed using a rhPDGF-BB-specific ELISA Kit (R&D System, Minneapolis, MN). Detected absorbance values were compared to a standard, as specified by the manufacturer’s instructions for determination of PDGF concentration. The amount of PDGF detected was normalized to the weight (mg) of the corresponding scaffold used.

### Migratory Property of Released Platelet-Derived Growth Factor

Migration of human dermal fibroblasts was evaluated using ORIS™ cell migration assay kit (Platypus Technologies, Madison, WI) to assess PDGF bioactivity. Briefly, fibroblasts treated with mitomycin C (Sigma-Aldrich, St. Louis, MO) were trypsinized and seeded into 96-well plates containing stoppers provided by the manufacturer and incubated at 37 **°**C and 5% CO_2_ overnight. The following day, stoppers were removed creating migration zones to which 100 μL of eluates collected at various time points was added and incubated for an additional 24 h. Freshly prepared 50 ng mL^−1^ PDGF and basal fibroblast media were used as positive and negative controls, respectively. Fibroblast migration was expressed as a fold change, compared to the migration elicited by the 50 ng mL^−1^ PDGF treatment. Studies were performed in triplicate in three independent experiments for three loading concentrations (2, 4, and 8 μg/mL).

### Statistical Analysis

Continuous data were expressed as means ± standard deviations. Differences among group means were analyzed using one-way analysis of variance (ANOVA). Tukey’s multiple comparison test was used to determine which means among a group of means were statistically different. Statistical significance was set at *α* = 0.05. All data were analyzed using GraphPad Prism (San Diego, CA, USA).

## Results and Discussion

The combination of various polymers has been shown to significantly improve the properties of the resulting composite [29, 30]. Combinatory polymer systems effectively allow for the modulation of scaffold strength, flexibility, biodegradation, and drug release kinetics, which are important parameters for wound dressing applications. However, the creation of unique composites can be limited by intrinsic chemical interactions between the constituent polymers. For example, a polymer precipitate was observed to form when mixing negatively charged Fb and positively charged CS; this observation is well-documented for molecules with strong electrostatic interactions under various solvent conditions [31–33]. Consequently, fabrication of a CS-Fb polymer composite scaffold required the use of a dual extrusion electrospinning technique, as previously described [34].

### Morphology of CS-PEO/Fb Nanofibers

**Fig. 2:**
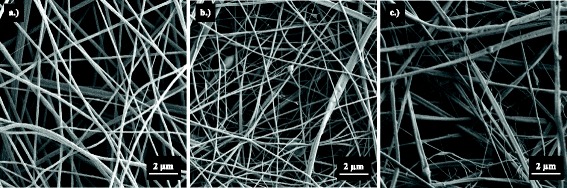
Representative FESEM micrographs of electrospun scaffolds. **a** CS-PEO, mean diameter 269.9 ± 68.4 nm. **b** CS-PEO/Fb, mean diameter 202.3 ± 113.2 nm. **c** Fb, mean diameter 351.1 ± 101.7 nm (*n* = 100)

Mean fiber diameters for CS-PEO, CS-PEO/Fb, and Fb scaffolds were 269.9 ± 68.4, 202.3 ± 113.2, and 351.1 ± 101.7 nm, respectively (Fig. 2). Interestingly, CS-PEO/Fb composite scaffolds exhibited thinner nanofibers than either the CS-PEO or Fb scaffolds. The decrease in fiber diameter size could be due to additional electrostatic forces generated by simultaneously charged polymer jets [35–37]. The repulsion of the polymer jets away from one another caused by these forces could therefore increase both the inter-spinneret distance and time of deposition. The resulting extended deposition time correlates with increased bending instabilities that are known to cause fiber elongation and thinner fiber formation.

### Surface Chemistry Characterization via ToF-SIMS and Water Contact Angle

**Fig. 3:**
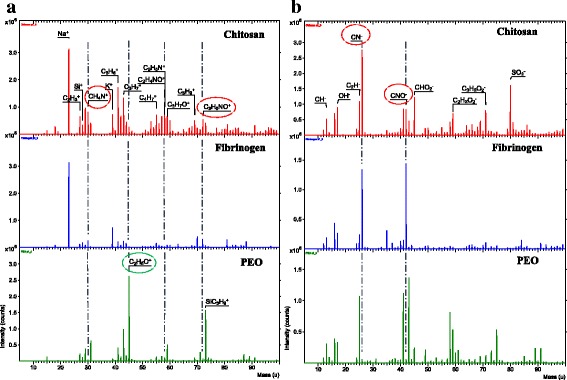
Representative **a** positive and **b** negative ion ToF-SIMS spectra for pristine CS (top), Fb (middle), and PEO (bottom). Circled ion fragments were utilized as the unique fingerprint to identify the compound

The ToF-SIMS spectra from CS, Fb, and PEO scaffolds are shown in Fig. 3. Normally, unique chemical fingerprint species can be used to distinguish individual polymer components. Due to the chemical similarities between CS and Fb, however, there was a number of nitrogen containing species (C_3_H_6_NO^+^, CH_4_N^+^, CN^−^, and CNO^−^) present in both spectra, making it difficult to distinguish the polymers from one another. However, when Fb was incorporated in the scaffold, the total number of CN^−^ and CNO^−^ species increased whereas the other fragments remained unchanged or decreased. Therefore, examining changes in CN^−^ and CNO^−^ fragment intensities allowed for the indirect detection of Fb. Similarly, the increase of C_2_H_5_O^+^ (45 m/z) ions was used to identify the ethylene oxide fragments of the PEO in the composite scaffolds.

**Fig. 4:**
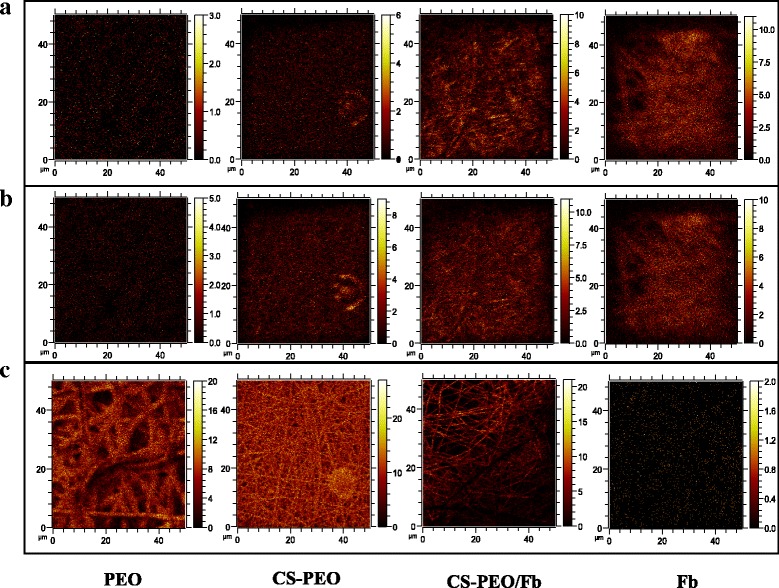
ToF-SIMS mapping of **a** CNO^−^, **b** CN^−^, and **c** C_2_H_5_O^+^ ion fragments on various scaffolds over a 50 × 50-μm raster area

To elucidate the presence and distribution of Fb components within the composite scaffolds, CNO^−^ and CN^−^ ions were mapped over a 50 × 50-μm raster of the pristine and composite scaffolds to obtain heat maps (Fig. 4a, b). The same mapping technique was performed on C_2_H_5_O^+^ to determine PEO distribution (Fig. 4c). Increased heat map intensity was correlated with higher polymer content of Fb or PEO. We observed that the intensities of CNO^−^, CN^−^, and C_2_H_5_O^+^ were uniformly distributed throughout the 50 × 50-μm raster area of the composite scaffolds. Due to the thickness of the scaffolds (93.8 ± 7.4 μm) and layer-by-layer fiber deposition, the surface composition is expected to be representative of the whole scaffold. Hence, the ToF-SIM data suggest that when CS-PEO/Fb nanofibrous scaffolds are fabricated using dual-spinneret electrospinning, individual components are deposited uniformly throughout the composite scaffold.

**Fig. 5:**
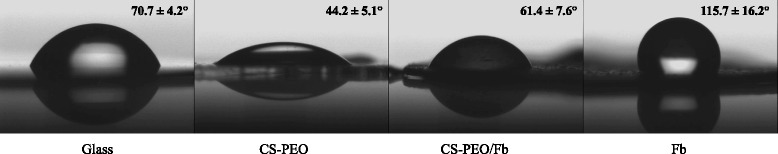
Behavior of deionized water on the surface of a glass substrate, CS-PEO, CS-PEO/Fb, and Fb scaffolds

Water contact angles of electrospun CS-PEO, CS-PEO/Fb, and Fb fibrous scaffolds were determined to be 44.2° ± 5.1°, 61.4° ± 7.6°, and 115.7° ± 16.2°, respectively (Fig. 5). Based on the water contact angles, CS-PEO and CS-PEO/Fb scaffolds were hydrophilic (> 90°), while Fb scaffolds were hydrophobic (< 90°). Water contact angles of CS-PEO/Fb composite scaffolds demonstrated surface characteristics that were between those of its components, which suggest that CS-PEO and Fb were homogenously deposited during fabrication. Surface characteristics of polymeric surfaces play an important role in the deposition of proteins and adhesion factors. Specifically, important bacterial adhesion factors are known to occur more readily on hydrophobic surfaces, which promote bacterial colonization [38]. As such, the hydrophilic nature of the CS-PEO/Fb might be beneficial in deterring bacterial attachment.

### Mechanical Uniaxial Tensile Strength of CS-PEO/Fb Scaffolds

**Fig. 6:**
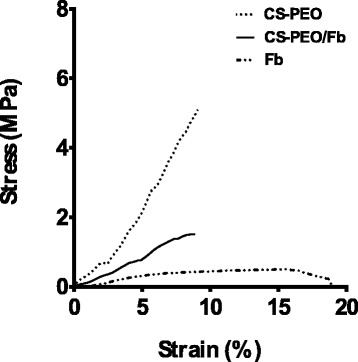
Stress-strain curves of CS-PEO, CS-PEO/Fb, and Fb scaffolds. Data are representative of ten independently produced scaffolds

Uniaxial tensile testing was performed on nanofibrous scaffolds with a mean thickness of 93.8 ± 7.4 μm. A summary of results is shown in Table 1, and corresponding stress-strain curves are shown in Fig. 6. Generally, mechanical properties of composite materials are correlated with its weakest constituent. Results suggest that Fb-only scaffolds would not have ideal mechanical properties for wound dressing applications. This emphasizes the importance of incorporating CS-PEO to obtain a more mechanically stable product.

**Table 1:** Uniaxial tensile properties of electrospun scaffolds (*n* = 10)

Scaffold	Young’s modulus (MPa)	Tensile stress (MPa)	Strain at break (%)
CS-PEO	78.12 ± 15.24*	7.27 ± 3.00*	9.88 ± 2.69
CS-PEO/Fb	18.84 ± 5.19	1.28 ± 0.38	8.25 ± 1.70
Fb	6.49 ± 1.89*	0.37 ± 0.10*	26.44 ± 8.58*

Comparisons made to CS-PEO/Fb scaffolds

*p < 0.05

### Water Vapor Transfer Rate of CS-PEO/Fb Scaffolds

**Fig. 7:**
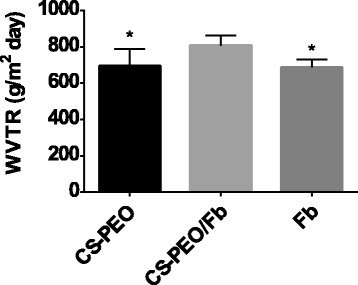
Water vapor transfer rates of the electrospun CS-PEO, CS-PEO/Fb, and Fb scaffolds. Error bars represent standard deviation (*n* = 6, *p < 0.05 when compared to CS-PEO/Fb scaffolds)

WVTR plays a vital role in maintaining an ideal humidity of the wound environment, which positively impacts cellular granulation and epithelization [39, 40]. Higher WVTR values correlate with rapid wound dehydration due to evaporation and exudation, which can adversely decrease body temperature and increase metabolism. Conversely, extremely low WVTR values correlate with exudate accumulation, inhibition of healing, and increased risk of infection. Lamke et al. reported the WVTR for normal skin as 204 ± 12 g m^−2^ day^−1^ and between 279 and 5138 g m^−2^ day^−1^ for injured skin, depending on the condition of the wound [41, 42]. For wound epithelialization and healing enhancement, an ideal WVTR of 2028.3 ± 237.8 g m^−2^ day^−1^ has been reported [43]. WVTRs of CS-PEO and Fb scaffolds were 693.2 ± 95.7 and 686.1 ± 44.17 g m^−2^ day^−1^, respectively. Although closer to the ideal WVTR than the pristine scaffolds, CS-PEO/Fb scaffolds (806.5 ± 56.1 g m^−2^ day^−1^) still fell below the ideal WVTR (Fig. 7). Manipulations to the physical parameters of the scaffold could improve WVTRs to better mimic reported ideal rates.

### In Vitro Cellular Viability of Human Dermal Fibroblasts

**Fig. 8:**
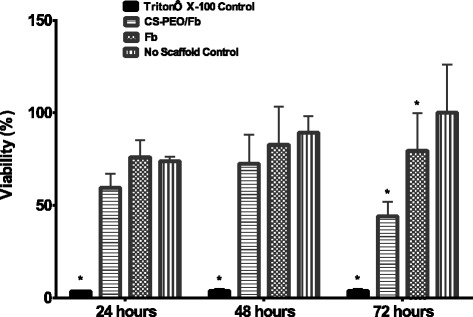
Comparison of human dermal fibroblast proliferation when exposed to Fb and CS-PEO/Fb electrospun scaffold (*n* = 9, *p < 0.05 comparison made to “no scaffold control” at each time point)

Chitosan-PEO/Fb scaffolds did not exhibit a significant proliferative effect on human fibroblasts during the initial 48 h (Fig. 8); yet, prolonged exposure at 72 h produced a statistically significant reduction in proliferation compared to the no scaffold control. The results suggest that there is inhibition of cellular proliferation exhibited by the scaffold, which may be cumulative over time.

The decreased proliferative capacity may be due to the degree of acetylation (DA) associated with CS, which has been shown to have strong cellular interactions through its positive charges [44]. Younes et al. demonstrated bladder carcinoma cells treated with CS (> 50% DA) significantly reduced viability after 24 h, suggesting CS cytotoxicity is directly related to its DA which can impact proliferation [45]. This effect may also be isolated to in vitro experimental conditions; previous studies have shown CS to be non-toxic in vivo, as its biodegradation products could be cleared through metabolic pathways [46]. Residual HFIP from the electrospinning process may also contribute to the observed cytotoxicity. However, the same cytotoxicity was not observed in the electrospun pure Fb scaffold, which was also prepared in HFIP. HFIP was likely eliminated from electrospun scaffolds during the fabrication process due to its high volatility. An observable decrease in cell viability would be evident if residual HFIP incorporated into the fiber system was being released during timed exposure to human dermal fibroblasts.

### LIVE/DEAD Assay and Cellular Attachment

**Fig. 9:**
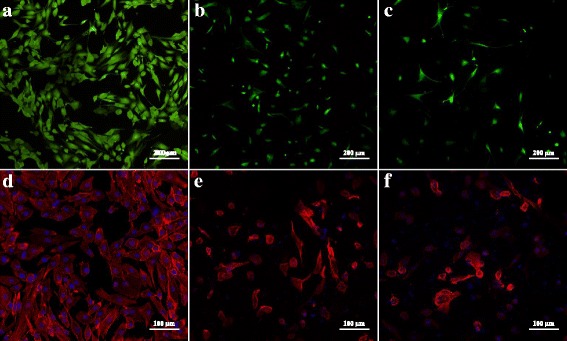
Confocal micrographs of fibroblasts seeded on **a**, **d** fibronectin-coated glass; **b**, **e** unloaded CS-PEO/Fb scaffold; and **c**, **f** PDGF-loaded (4 μg/mL) CS-PEO/Fb scaffolds. **a–c** Cells were stained with LIVE/DEAD™: live cells (green), dead cells (red). **d–f** Cells were stained with DAPI (blue) and phalloidin (red)

Figure 9a–c shows the distribution and viability of dermal fibroblasts seeded on the surface of fibronectin-coated glass, unloaded CS-PEO/Fb scaffolds, and PDGF-loaded (4 μg/mL) CS-PEO/Fb scaffolds. After 24 h in culture, minimal dead cells were observed in comparison to the live cells. No discernable differences between PDGF-loaded and unloaded scaffolds were noted.

Fibrinogen serves as an important mediator of cellular attachment and growth during wound healing. Phalloidin stains cellular actin filaments allowing for visualization of fibroblast attachment. Figure 9c, d shows the actin filament morphology of the fibroblasts on the surface of fibronectin-coated glass, unloaded CS-PEO/Fb scaffolds, and PDGF-loaded CS-PEO/Fb scaffolds. Overall, normal fibroblast morphology was observed, with the exception of some rounder cells being present on the scaffold samples. The changes in morphology may be attributed to the presence of CS, as shown in previous studies [47]. Alternatively, the differences could be due to the small porosity of the scaffold and dimensionality of the fiber surface in comparison to the flat surface of the control.

### In Vitro Degradation of Electrospun CS-PEO/Fb Scaffolds

**Fig. 10:**
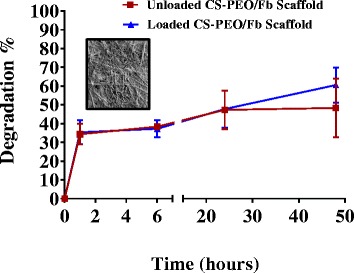
Degradation of electrospun unloaded CS-PEO/Fb scaffolds and PDGF-loaded (8 μg/mL) CS-PEO/Fb scaffolds. Insert: FESEM micrograph of CS-PEO/Fb scaffold after 48 h of incubation (*n* = 6)

Scaffold degradation was evaluated by obtaining the dry weight of the scaffold after incubation in FBM for up to 48 h (Fig. 10). The scaffolds degraded at a linear rate after an initial burst release of material within the first hour. Weight loss in the initial hour was likely due to the solubility of PEO in aqueous solutions [16], and the subsequent weight loss was likely due to Fb and CS release. This highlights a unique biphasic degradation profile that could be beneficial for the delivery of bioactive molecules. Concurrently, CS-PEO/Fb scaffolds were shown to maintain their substructure for a minimum of 48 h (Fig. 10: insert), and the addition of 8 μg/mL of PDGF did not alter the scaffold degradation properties.

### In Vitro Release of PDGF

**Fig. 11:**
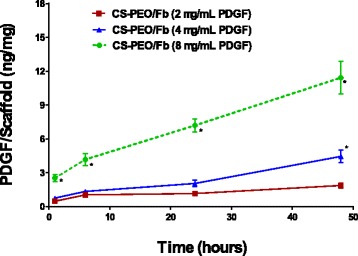
PDGF is released from CS-PEO/Fb scaffolds for up to 48 h in a dose-dependent manner (*n* = 10, *p < 0.05 when compared to 2 μg/mL of PDGF dosage at each specific time point, ^#^*p* < 0.05 when compared to 4 μg/mL of PDGF dosage at each specific time point)

Cumulative releases of PDGF after 48 h of incubation were 1.8 ± 0.7, 4.4 ± 1.8, and 11.4 ± 4.8 ng of PDGF/mg of scaffold for initially incorporated concentrations of 2, 4, and 8 μg/mL of PDGF, respectively. The results demonstrated that PDGF was released from the scaffolds in a dose-dependent manner (Fig. 11).

During the in vitro degradation, CS-PEO/Fb scaffolds exhibited a unique biphasic release profile, with 21.6 ± 0.1% of the total eluted PDGF being detected in the first hour followed by linear release kinetics. This observation was thought to be a result of the combined polymers and highlighted the functional importance of Fb as a reservoir for the sustained release of biologically critical molecules. Growth factors (e.g., PDGF/VEGF, FGF, and TGF-β families) have been shown to bind the Fb heparin domain with high affinity and have been reported to retain their bioactivity for over 7 days [48–50].

### Effects of Released PDGF on Fibroblast Migration

**Fig. 12:**
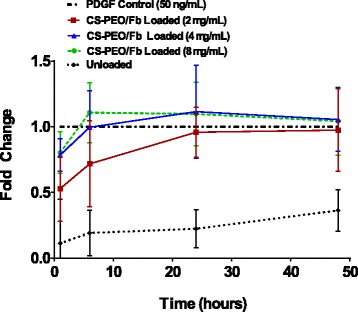
Human dermal fibroblast migration after 24 h exposure to scaffold eluates acquired at 1, 6, 24, and 48 h in FBM (*n* = 8, *p < 0.05 when compared to unloaded CS-PEO/Fb scaffold at each specific time point)

Fold differences in fibroblast migration in response to the released PDGF eluates collected from the scaffolds at various time points are shown in Fig. 12. Previously published reports have demonstrated that fibroblast attachment and migration rates are affected by PDGF in a dose-dependent manner [51, 52]. For example, Gamal et al. showed when at least 50 ng/mL PDGF was delivered locally, adherent fibroblast migration was increased [52]. In addition, Thommen et al. reported that the distance of migration was significantly increased when exposed to PDGF concentrations greater than 10 ng/mL [53]. Therefore, the biological activity of the scaffold-eluted PDGF was measured by its ability to induce migration and was normalized to a single 50 ng/mL PDGF dose.

Eluates collected from electrospun scaffolds, which were loaded with 4 or 8 μg PDGF/mL polymer solutions, demonstrated similar levels of fibroblast migration when compared to a single 50 ng/mL treatment. The data suggest that PDGF delivered by electrospun nanofibers can be equally effective in promoting fibroblast migration as a single application of PDGF. Additionally, the migration elicited by the scaffold-released PDGF was sustained for 48 h. Sustained delivery of PDGF eliminates the need for daily applications, which is an advantage over commercially available treatments such as Regranex®.

Fibroblast migration reached a maximum when cells were treated with the 24-h eluates from each of the PGDF-loaded scaffolds. Notably, when fibroblasts were treated with the 6-h eluates from the 4 and 8 μg PDGF/mL electrospun scaffolds, the same level of maximum migration was achieved. This suggests that the rate of migration increased in response to increased PDGF loading. Additionally, 4 and 8 μg/mL PDGF-loaded scaffolds might be able to elicit fibroblast migration potentials beyond the detection limits of the current assay, which can only assess the endpoint of migration, but not the migration rate. Finally, results demonstrated that eluates from unloaded CS-PEO/Fb scaffolds were also capable of eliciting a linear increase in migration over the same elution time points, albeit less effectively than the PDGF-loaded scaffolds. This result is most likely mediated by the ability of fibrinogen to enhance fibroblast migration [54, 55]. In general, PDGF-loaded composite scaffolds significantly improved in vitro migration, compared to the individual components of the scaffold.

## Conclusion

The versatility of electrospinning allows for the combination of advantageous properties of various polymers and proven bioactive molecules. We utilized this technique to fabricate unique CS-PEO/Fb scaffolds with the ability to release biologically active PDGF over 48 h. This study evaluated the chemical and physical properties of the nanofibrous scaffolds including (1) morphology, (2) physical and mechanical properties, (3) in vitro scaffold degradation, and (4) the ability to release functional PDGF in a dose- and time-dependent manner. Electrospun CS-PEO/Fb scaffolds demonstrated nanoscale morphological properties that are conducive to wound dressing applications, as well as an adequate WVTR, mechanical stability, and a unique biphasic PDGF delivery profile. Furthermore, PDGF maintained its biological function throughout the electrospinning process, and when combined with the natural, chemotactic properties of Fb elicited dermal fibroblast migration that is functionally equivalent to a single 50 ng/mL dose of PDGF. Overall, these results highlight the potential of CS-PEO/Fb scaffolds as a viable wound dressing capable of delivering bioactive PDGF to enhance fibroblast recruitment and wound healing.

## Abbreviations

CS: Chitosan
DA: Degree of acetylation
DMSO: Dimethyl sulfoxide
ECM: Extracellular matrix
EMEM: Eagle’s minimum essential media
Fb: Fibrinogen
FBM: Fibroblast blast media
FESEM: Field emission scanning electron microscope
FGF: Fibroblast growth factor
HFIP: 1,1,1,3,3,3-Hexafluoroisopropanol
PDGF: Platelet-derived growth factor
PEO: Polyethylene oxide
PLGA: Poly(lactide-co-glycolide)
TGF-β: Transforming growth factor beta
ToF-SIMS: Time-of-flight secondary ion mass spectrometry
VEGF: Vascular endothelial growth factor
WVTR: Water vapor transfer rate
